# Connection to the land as a youth-identified social determinant of Indigenous Peoples’ health

**DOI:** 10.1186/s12889-018-6383-8

**Published:** 2019-02-11

**Authors:** Laurie-Ann Lines, Yellowknives Dene First Nation Wellness Division, Cynthia G. Jardine

**Affiliations:** 1 Health Promotion and Socio-behavioural Sciences PhD Student, School of Public Health, University of Alberta, Edmonton, Canada; 2 Collectively representing all staff members of the Yellowknives Dene First Nation Wellness Division who were involved in the design, implementation, analysis and interpretation of the research, Yellowknive, Canada; 3 Tier 1 Canada Research Chair in Health and Community, Faculty of Health Sciences, University of the Fraser Valley, Chilliwack, Canada

**Keywords:** Indigenous, Youth, Land connection, Health, Cultural skills, Social determinants, Structural determinants, Strength-based, First Nations

## Abstract

**Background:**

Social determinants of Indigenous health are known to include structural determinants such as history, political climate, and social contexts. Relationships, interconnectivity, and community are fundamental to these determinants. Understanding these determinants from the perspective of Indigenous youth is vital to identifying means of alleviating future inequities.

**Methods:**

In 2016, fifteen Yellowknives Dene First Nation (YKDFN) youth in the Canadian Northwest Territories participated in the 'On-the-Land Health Leadership Camp'. Using a strength- and community-based participatory approach through an Indigenous research lens, the YKDFN Wellness Division and university researchers crafted the workshop to provide opportunities for youth to practice cultural skills, and to capture the youth’s perspectives of health and health agency. Perspectives of a healthy community, health issues, and health priorities were collected from youth through sharing circles, PhotoVoice, mural art, and surveys.

**Results:**

The overall emerging theme was that a connection to the land is an imperative determinant of YKDFN health. Youth identified the importance of a relationship to land including practicing cultural skills, Elders passing on traditional knowledge, and surviving off the land. The youth framed future health research to include roles for youth and an on-the-land component that builds YKDFN culture, community relations, and traditional knowledge transfer. Youth felt that a symbiotic relationship between land, environment, and people is fundamental to building a healthy community.

**Conclusion:**

Our research confirmed there is a direct and critical relationship between structural context and determinants of Indigenous Peoples’ health, and that this should be incorporated into health research and interventions.

## Background

In recent years there has been growing recognition that ‘health’ is more than an individualistic, biomedical concept - it is also determined by social circumstances and contexts. These ‘social determinants of health’ involve the conditions under which people live and work, and include diverse factors such as income, education, stress, social marginalization, racism and food security [1]. Because they influence the health status of individuals and groups, deficits in the social determinants of health are considered the major underlying reasons for health inequities and inequalities between populations [2].

Although this is considered a relatively ‘new’ approach to health and wellbeing in the Western world, Indigenous Peoples in Canada and elsewhere have always known that health is a holistic concept that extends beyond individual behaviours and genetics [3]. Social determinants of health in an Indigenous context also include unique structural (or foundational) determinants such as history, political climate, economics and social contexts. Relationships, interconnectivity, and community are fundamental to these determinants [4].

Yet, ironically, simply being Indigenous is considered a significant social determinant of health in Canada [5]. Indigenous people in Canada rank lower in almost every determinant of health than do non-Indigenous Canadians. In 2011, 29% of Indigenous adults aged 25 to 64 had less than a high school education (with the most alarming rates of almost 50% being in First Nations on reserve and Inuit populations), compared to nearly 12% of the non-Indigenous population [6] (in Canada, Indigenous Peoples refers to First Nations, Métis and Inuit Peoples). Indigenous Canadians are almost three times more likely to experience food insecurity than their non-Indigenous counterparts, with more than 230,000 Indigenous youth aged 12–17 estimated to live in households with moderate to severe food insecurity [7]. These deficits are reflected in multiple adverse health outcomes, including, but not limited to, elevated rates of: infant and young child mortality, infectious diseases, malnutrition, tobacco use, accidents, interpersonal violence, homicide, suicide, obesity, cardiovascular disease, diabetes, and diseases caused by environmental contamination [8]. For example, the age-standardized rate for diabetes is 17.2% for First Nations individuals living on-reserve, compared to 5.0% for other Canadians; diagnosis usually occurs at a younger age than non-Indigenous individuals [9]. Smoking for youth aged 12 to 24 is two times more likely in an Indigenous population, excluding reserves, than within the non-Indigenous population [6]. The prognosis for those who suffer ill-health is also worse; First Nations people with cancer have a significantly poorer 5-year survival than their non-Indigenous peers [10].

The continued effects of colonialism are considered to be the most important determinant of health for Indigenous Canadians [11]. Although colonialism has been adapted into many present forms, the symptoms of colonialism largely stem from the devastations of “Indian Residential School systems” occurring in Canada from 1892 to the mid 1990’s to fulfill the Indian Affairs Deputy Superintendent’s goal to “… get rid of the Indian problem … until there is not a single Indian in Canada…” [12] (p. 3). The residential schools were a deliberate system of forced assimilation that often started with forcible removal of children from their homes, and ended in many cases of physical, emotional, and sexual abuse including slavery, spread of infectious diseases, violent punishment for speaking an Indigenous language, and death [12]. Many students battled the destruction of their culture, language and identity and were denied cultural opportunities to develop parenting skills, speak their language, take part in spiritual ceremonies, or practice cultural activities such as harvesting off the land [12]. The intergenerational consequences of trauma from the attempted cultural genocide are still manifested in the health status of Canadian Indigenous populations. Despite narrowing disparities in mental and physical health status, Indigenous Peoples continue to suffer an increased burden of illness, and to be disadvantaged relative to non-Indigenous people in Canada on numerous markers of morbidity and mortality, including socioeconomic deprivations, inequalities entrenched in education and healthcare systems, and experiences of violent victimization [13, 14]. Health issues faced by Indigenous Peoples are a multiplicative product, rather than additive sum, of cultural wounds affecting the entire community and ways of life [15]. The social determinants of health related to these historical contexts have largely been accepted as one of the key etiologic factors underlying high rates of illness, disease, and mortality in Indigenous populations [16].

Addressing inequities resulting from shortfalls in the social determinants of health is an ongoing problem, particularly for Indigenous Peoples. A research approach that has gained support is changing the focus to community strengths (“what has worked in the past and what is the most appropriate community vision for future success”) as opposed to the more traditional focus on problems (“why and where the community has failed”) [17] (p. 44). Strength-based research is premised on empowering everyone involved in the research (community members, academic researchers, policy-makers, etc.) to create social change [18]. This is consistent with viewing social determinants of health as ‘health promoting’ factors that are influenced by political ideologies, public policies, societal recognition and outrage, and the ability of individuals and communities to effect change [19]. Using a health promotion lens shifts the focus from a pathogenic approach that emphasizes factors related to disease and illness to a salutogenic model that stresses positive, salutary factors that support people’s health and well-being [20, 21].

Exploring determinants of health from the perspective of Indigenous youth is critical to identifying and changing the conditions underlying inequities that impact individuals, communities and nations in the next generation [22]. With almost half (46%) of Canadian Indigenous people being under the age of 25 [23], Indigenous youth are the key to future change. They also hold a special place in their communities and collectives: “they embody the past through our teachings, they experience the present, and they hold our dreams for the future. Their individual identities ensure collective continuity” [24] (p. 65). Identifying the social and structural determinants of health from the perspective of Indigenous youth is important because it is a “contemporary re-articulation of traditional egalitarian practices that recognized the central role of youth in the health and vitality of the community” [25] (p. S21). As Indigenous youth are known to “espouse a broader approach to health that considers the linkages between culture, identity and health” [26] (p. 87), their contributions are potentially extremely valuable in shaping Indigenous health programs in Canada.

Many Indigenous scholars emphasize the importance of including youth’s health perspectives when capturing a community’s holistic health perspective [27–30]. However, in North America, Indigenous youth’s abilities are typically underestimated or underutilized by the ‘Western’ public and research community, where adults usually practice ‘adultism’ or hold power over youth [31]. This colonial view denies youth’s agency to create change [30], such as influence and effect health services, and opposes an Indigenous perspective. In First Nations communities, youth were traditionally called on as leaders to voice opinions in decision making, act as role models to increase positive outcomes for the next generation, shelter responsibilities for the community, and act on behalf of the community in addressing issues such as health, communication, justice, and food security [27, 28].

In this article, we discuss an asset or strength-based approach to exploring concepts of health and healthy communities through the eyes of Indigenous youth in northern Canada as a precursor to awareness of and change in social and structural determinants of Indigenous health. The research questions that informed our investigation were:

- What are the perspectives of Yellowknives Dene First Nation (YKDFN) youth on health, health issues and health priorities?
- How do YKDFN youth understand the factors that determine their ‘health’ or ‘being healthy’ within their community? What factors do they think are important?
- What are the perspectives of YKDFN youth on their role in future health research?

## Methods

### Methodology

An integral component of research partnerships and reciprocal knowledge exchange between communities and universities is a participatory approach with elements of trust and relationship building [32]. To accentuate community strengths and Indigenous knowledge, we used a community-based participatory research (CBPR) methodology through an Indigenous research lens. CBPR is a collaboration between researchers and community participants through sharing knowledge and relevant lived experiences to promote social change [33, 34]. An Indigenous methodology is based on relationality and is best carried out by an Indigenous researcher who carries forward these lifelong relationships [35]. An Indigenous methodology is informed by Indigenous knowledge systems and challenges former colonial descriptions in reconstructing knowledge to include an Indigenous perspective [36]. Our project employed a decolonizing CBPR approach [37] that was grounded by Indigenous community relations to challenge the oppressive acts of colonization by reconstructing concepts of health to include Indigenous knowledge.

### Relationships

While first author Laurie-Ann Lines was the academic researcher for this project, she is also a member of the YKDFN. She conducted the research as part of her graduate program dissertation, under the supervision and guidance of senior author Dr. Cindy Jardine. Dr. Jardine, in her capacity as a university professor and researcher, has previously led many CBPR projects involving Indigenous youth and health promotion initiatives in the YKDFN. Over the past decade, Laurie-Ann has worked, volunteered, and partnered with various divisions in the YKDFN, serving YKDFN children, youth, adults, and Elders. The research was conducted in partnership members of the YKDFN Wellness Division, who were active in the planning, implementation, and interpretation of the research, including assisting in workshop organization and participant recruitment, analysis of results and manuscript preparation. Additional research partners included two community research assistants, (who assisted the researchers in notetaking, observations, and monitoring research activities) and traditional knowledge and cultural resource workers (who assisted in running the on-the-land workshop, provided observations, and shared their knowledge with the youth). The YKDFN Wellness Division viewed this research as providing information for subsequent community health research programming, strategies, and policies.

### Study design and participants

The research was based in the two YKDFN communities of Ndilo and Dettah, and at a YKDFN traditional on-the-land camp site, all located near Yellowknife in the Canadian Northwest Territories. The First Nation has a population of approximately 1500 members, with about 600 currently living in Ndilo and Dettah, and the remainder primarily living in Yellowknife. Approximately 44% of members are under the age of 25 [38].

Over the period of a week (Monday through Friday) in August 2016, the exploration of youth’s health perspectives was interwoven with leadership skill development and YKDFN cultural camp activities through the YKDFN Youth Health Leadership On-the-Land Workshop. On Monday, the youth conducted research activities in the communities. From Tuesday to Friday the youth completed remaining research activities at the on-the-land camp. The YKDFN Wellness Division organized the camp activities where youth practiced and further developed YKDFN cultural skills, such as traditional harvesting. The on-the-land camp was held at a location removed from city and community life, where the YKDFN Wellness Division holds an annual summer cultural camp for youth. The YKDFN Wellness Division recruited participants through their existing networks and selected youth based on their age, availability, and community recommendations (such as including a variety of family lineages and at-risk youth). The fifteen youth participants, aged 13–18 years, were all of YKDFN descent.

We (the researchers and community partner) sought to understand youth’s perspectives of: 1) interest in health research; 2) health meaning, issues, and priorities; 3) tailoring healthy information and research to youth; 4) their role in future health research; and 5) their role in addressing health issues within their community. We used a mixed methods approach that consisted of two quantitative surveys (a short electronic ‘clicker’ polling survey and a longer iPad survey) and multiple types of qualitative data collection and analysis strategies including PhotoVoice, mural art, a modified ‘nominal group’ technique, sharing circles, observations and field notes, and personal reflections. Research projects with non-linguistic methods increase accessibility and illustration of further forms of understanding [39]. The two surveys were employed to better understand frequency of responses amongst study participants, realizing that the small sample size precludes any valid statistical analysis and that the results are not necessarily transferable to other populations.

The short survey captured interest in health research and used electronic ‘clicker’ polling equipment. The survey involved five multiple-choice questions and was conducted on the first day of the workshop. This was meant to engage youth in the research process anonymously and set the participatory tone. One question was designed to collect a general idea of youth’s interest in being involved in research and learning research skills.

The PhotoVoice project also occurred on the first day. Youth were divided into two groups for each community of Ndilo and Dettah and each given a digital camera or videorecorder. Youth walked around their community to capture health issues and priorities in photos or videos. PhotoVoice projects have been appreciated by youth as a means of sharing their voice and opinions through photos they have taken, edited, and narrated [40, 41]. The PhotoVoice method developed by Wang and Burris [42] presents a foundation for group participatory analysis in three stages: 1) selecting photographs that are most representative, 2) providing background for the photographs through stories, and 3) recognizing issues or themes. The group analysis allows for participants to voice their own distinct experiences jointly, so that participants’ collective vision and voices can surface about a particular subject [42]. Analysis was done in groups of 3–6 participants, and the discussions were facilitated by the researchers and research assistants. The collective analysis was guided by the SHOWeD process (i.e. What do you **S**ee here? How does this relate to **O**ur lives? **W**hy does this concern or priority exist? What can we **D**o about it?) [43]. The discussions were audio recorded and the selected photographs were saved during the discussion onto the researcher’s computer file to correspond to the youth’s analysis.

The mural project was used to broaden and clarify youth’s health perspectives through defining a healthy community. As two groups representing each YKDFN community, youth were asked to brainstorm what makes a community healthy. The discussions were led by the researchers and YKDFN research assistants. Youth had free reign to draw images, design the murals, and organize images. Youth sketched most of their images on the murals on Monday and finished painting and analyzing their murals at the on-the-land camp. Art methods have previously been used to direct the analysis process by presenting participants’ perspectives in a metaphoric format [39]. The images in the mural project were jointly analyzed by youth using a consensus group format called the ‘nominal group’ technique, where participants form an idea, discuss in a group, then come to a consensus [44]. Led by the principal investigator, youth reflected individually on the entire mural, wrote down elements of a healthy community the mural conveyed, and discussed their answers. To assist reaching consensus, youth were given the same number of stickers (approximately 10) to place beside the characteristic they believed was most representative of a healthy community (they could place it on more than one). The elements of a healthy community with the most votes were considered representative. This analysis process was audio recorded and recorded in writing on ‘sticky pads’ and flip chart paper.

The longer survey was conducted using iPads and created using Qualtrics® software. The survey was originally created by a partnering Inuit youth case-study research group led by Dr. Chris Furgal at Trent University. It was adapted for the YKDFN community context and included local issues such as arsenic contamination (from the former Giant Mine gold mining operation near Ndilo), activities such as Dene games, and community organizations (The YKDFN Youth Health Survey, on health information, health research priorities and issues, and youth’s health ratings is available from the authors on request). The survey consisted of Likert scale questions and some open-ended responses. The survey had nine demographic questions and 22 questions on health information, rating individual health, rating other youth’s health, concern about health issues, pride in health, health related community programming, participating in future health research and surveys, and prioritizing health issues. Participant responses were anonymous. The questions in the survey were analyzed using Qualtrics® software, which categorically analyzed the data and created relative frequency statistics. The responses from the open-ended questions will be used for future community programming and research.

The sharing circles were intended for youth to further discuss health issues and youth involvement in future health research and community health initiatives. Traditional sharing circles, varying by First Nation, are described generally as a method that offers time and space for each participant in the circle, including ancestors, to share their opinions and story in their own style [45]. Similar to Traditional circles, there was a shared expectation of non-judgmental active listening, respectful behavior, and non-interference [35]. The circle questions also incorporated kinesthetic movement and visuals, to try to stimulate further understanding and discussion of the health topic [46]. Three sharing circles of 4–6 youth took place on-the-land for about 45 min each. Participants wrote answers both on paper to show the group and discuss, and on ‘sticky paper’ to put up on a board for everyone to see and discuss. Participants shared consecutively, so that their ideas built upon each other, similar to Wilson’s ‘talk circle’ method [35]. The discussions were audio recorded and by a research assistant on flip chart paper.

Laurie-Ann Lines also used her own observations, daily personal reflections, field notes, and debriefing perspectives of other research assistants involved to further nuance the understanding and interpretation of youth’s perspectives. Indigenous researchers recognize inner knowledge as part of the knowledge continuum in constructing data and understanding relational referencing [46]. Throughout the research, Laurie-Ann was directed by: the community protocols set forth by the YKDFN Wellness Division; guidance and advice from her family, Elders, and traditional knowledge holders in the community; and the Dene Laws. There are many Dene laws that hold shared Dene values, which are usually taught throughout a lifetime by Elders, knowledge holders, and the community through oral stories, observation, and experiential learning. Some Dene Laws, which have been translated into English, abbreviated, and written down are: Share what you have; Help each other; Love each other as much as possible; Be respectful of Elders and everything around you; Be polite and don’t argue with anyone; Young girls and boys should behave respectfully; Pass on the teachings; Be happy at all times.

## Results

The research results are presented as collective findings to recognize that the researchers could not have come to these conclusions without the participation of the YKDFN youth participants, YKDFN research assistants, YKDFN traditional knowledge camp resource workers, and YKDFN Wellness Division. However, although these people were collaboratively involved in the planning, data collection, and analysis stages of this research, any misrepresentations of the YKDFN youth’s perspectives or of the YKDFN community are of the lead author and researcher, Laurie-Ann Lines.

### Youth identifying the importance of a relationship to the land

Youth-identified health issues in photos and videos included littering, pollution, smoking, alcohol and drugs, arsenic contamination, and unsafe areas (like unfinished construction sites or infrastructure). Identified health priorities were pathways, garden and greenhouse, youth involvement, sports (including Dene games), community gatherings, Elders and culture, and nature/the land.

**Fig. 1:**
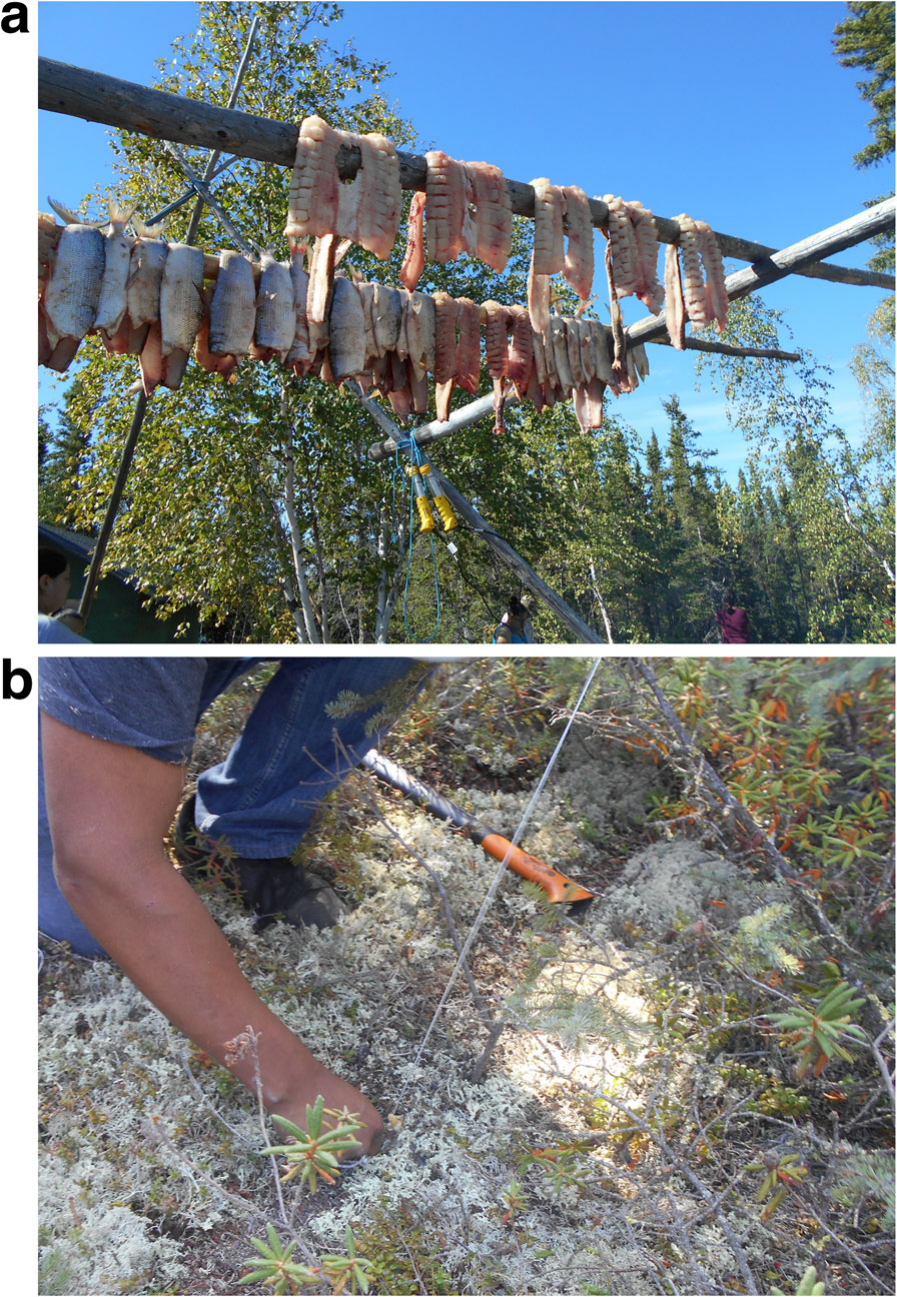
**a** and **b:** Youth’s PhotoVoice images denoted as ‘surviving off the land’

**Fig. 2:**
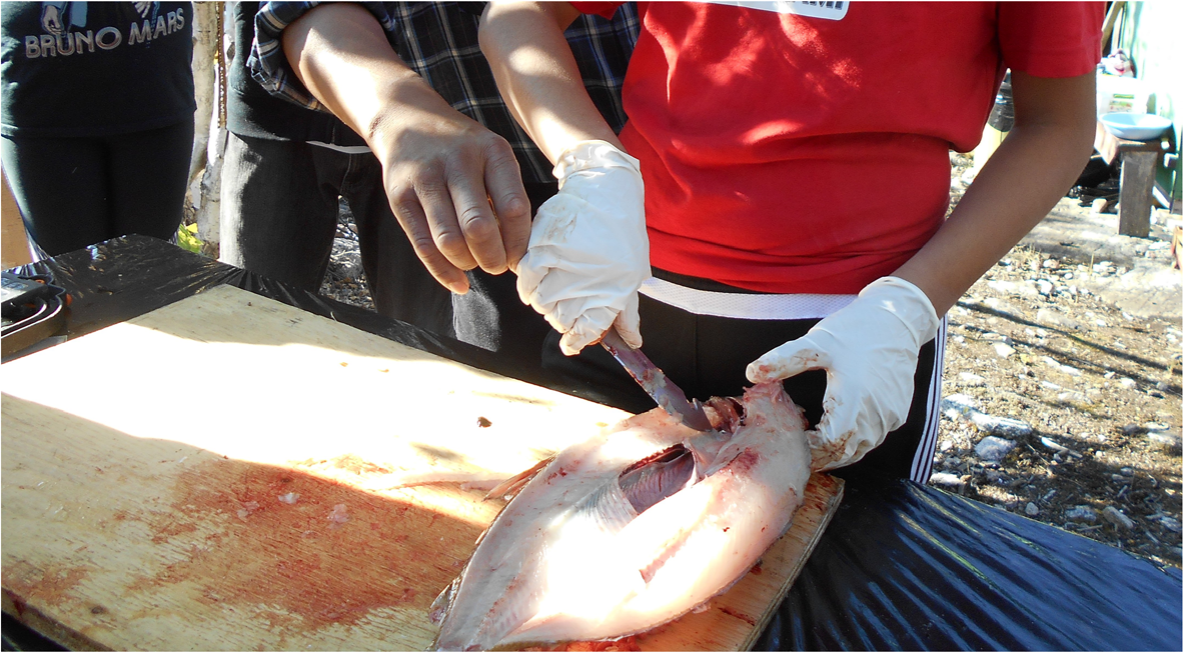
Youth’s PhotoVoice image denoted as ‘traditional knowledge’

However, after capturing images in the communities, the youth thought it was imperative to also have photos taken on-the-land depicting health priorities to fully express their concepts of health, even though this was not originally part of the PhotoVoice project. The youth emphasized the land-health relationship in photos and videos that showed: surviving off the land (Fig. 1), learning and passing on traditional knowledge (Fig. 2), cultural camp, practicing cultural skills, understanding YKDFN history, gathering and preparing food, being out on-the-land, and working together. Activities that promoted a connection to the land were considered health priorities by the youth.

The youth were purposeful in taking the photos. For example, the youth took a photo immediately following a hunting trip to capture their excitement of being able to provide traditional food and survive off the land. The youth captured many photos of the traditional knowledge camp resource workers using Indigenous teaching methods to pass on traditional knowledge. They identified teaching and learning traditional knowledge on-the-land as a health priority. Figure 2 displays one photo of passing on traditional knowledge where the youth practiced preparing fish alongside the traditional knowledge holder, while other youth observed and assisted. The youth wanted to depict actively being connected to the land by being respectful to animals and plants, traditionally harvesting and preparing foods, and practicing their cultural skills (Figs. 1 and 2).

Youth’s answers (*n* = 9) to questions on the iPad survey indicated pride in the health of youth in their community. The youth were proud of their culture (89%), practicing cultural activities (89%), and eating wild and traditional foods (78%) in relation to health. More than half of the total participants felt a positive link between culture (including on-the-land cultural activities) in relation to their health.

In the sharing circles, the youth’s discussions of addressing issues were largely based on the use of cultural camps and building on community assets. They felt other YKDFN youth need to know “our culture” and that “their issues are actually issues.” They also felt other youth “need to come [to culture camps] to remember the Dene laws.” Their discussions were representative of community issues and solutions, as echoed in community feedback, and showed gaps in addressing issues. This was noted in one of Laurie-Ann’s daily reflections, where she wrote from a community member’s perspective:

*I am surprised by how accurate the kids were in the issues they feel are facing them, because those are realistic issues facing the community. We [as adults] are the ones supposed to be facing them and dealing with them, so that the youth don’t feel them as strongly.* (Self-reflection, August 18, 2016).

### Youth perspectives on agency and future health research

In the electronic polling ‘clicker’ survey we asked youth “How interested are you in receiving research training and being involved with projects as a researcher?” All 13 youth were at least ‘somewhat interested’ in receiving research training and being involved with projects as a researcher, with 46% of participants being very or extremely interested.

From the debriefing discussions with other researchers, we concluded that the clicker survey was a starting point to engage youth in research and distinctly set a tone for youth participation, as captured in Laurie-Ann’s field notes:

*The other research assistants also noticed youth seeing their involvement as valuable because they were excited to contribute [their perspective]. Youth mentioned they want to lead a clicker survey during the youth’s family presentations.* (Field notes, August 15, 2016).

When youth explained their images in the PhotoVoice discussion, they were often metaphoric of a story or perspective, including their perceived agency and role in addressing health. For instance, one youth explained her ‘priority’ photo that depicted another youth taking pictures with a camera:

Youth 7: *It’s behind the scenes… [Youth should be involved in health], because the adults can’t do this all by themselves. Because we’re more active, [for example,] we took [these] pictures.*

In sharing circles of 3–5 participants, youth (*n* = 12) discussed health issues, communication of health information, and youth’s involvement in research and addressing health issues. Through collective exercises in the sharing circles, youth started to discuss concepts more in depth as noted in the abbreviated excerpt below:

Laurie-Ann: *If you were planning a health research project, what topic would you pick?*

Youth 1: *Garbage in the water.*

Youth 2: *Suicide.*

Youth 3: *Bullying.*

Laurie-Ann: *What are you picturing?*

Youth 3: *A camp.*

Laurie-Ann: *Are you thinking on-the-land?*

Youth 3: *Yes.*

Laurie-Ann: *What activities would you like to be involved in to do that research?*

Youth 2: *A trust exercise.*

Laurie-Ann: *Do you want to come up with those exercises?*

Youth 2: *Yeah.*

Laurie-Ann: *What other activities would you like?*

Youth 5: *[To] be a worker.*

Youth 2: *Teach more [cultural skills].*

Youth envisioned the next health research initiatives as taking place on-the-land to provide opportunity to continue their cultural practice, leadership skill development, and healthy living. Youth started to picture many roles for themselves in future health research initiatives, from planning the camp to facilitating a camp as a worker. In another sharing circle, youth participants similarly expressed their interest in being involved in the next youth health research initiatives through helping to plan topics and activities (such as designing surveys), advertising, working as camp assistants, and putting together communication products such as slideshows and movies on health. The youth saw it necessary that any future health research projects or health programs must have an on-the-land component, suggesting a series of on-the-land cultural camps (Laurie-Ann’s Field notes, August 15, 2016).

### Youth ‘voicing’ health as a symbiotic relationship of land and people

Youth had many opportunities to ‘voice’ their opinions of health during activities that were youth led, such as the mural creation and PhotoVoice project. Youth practiced and built their leadership and critical thinking skills through discussion, which propelled their further investigation into factors that determine ‘health’ and ‘being healthy’ within a community.

**Fig. 3:**
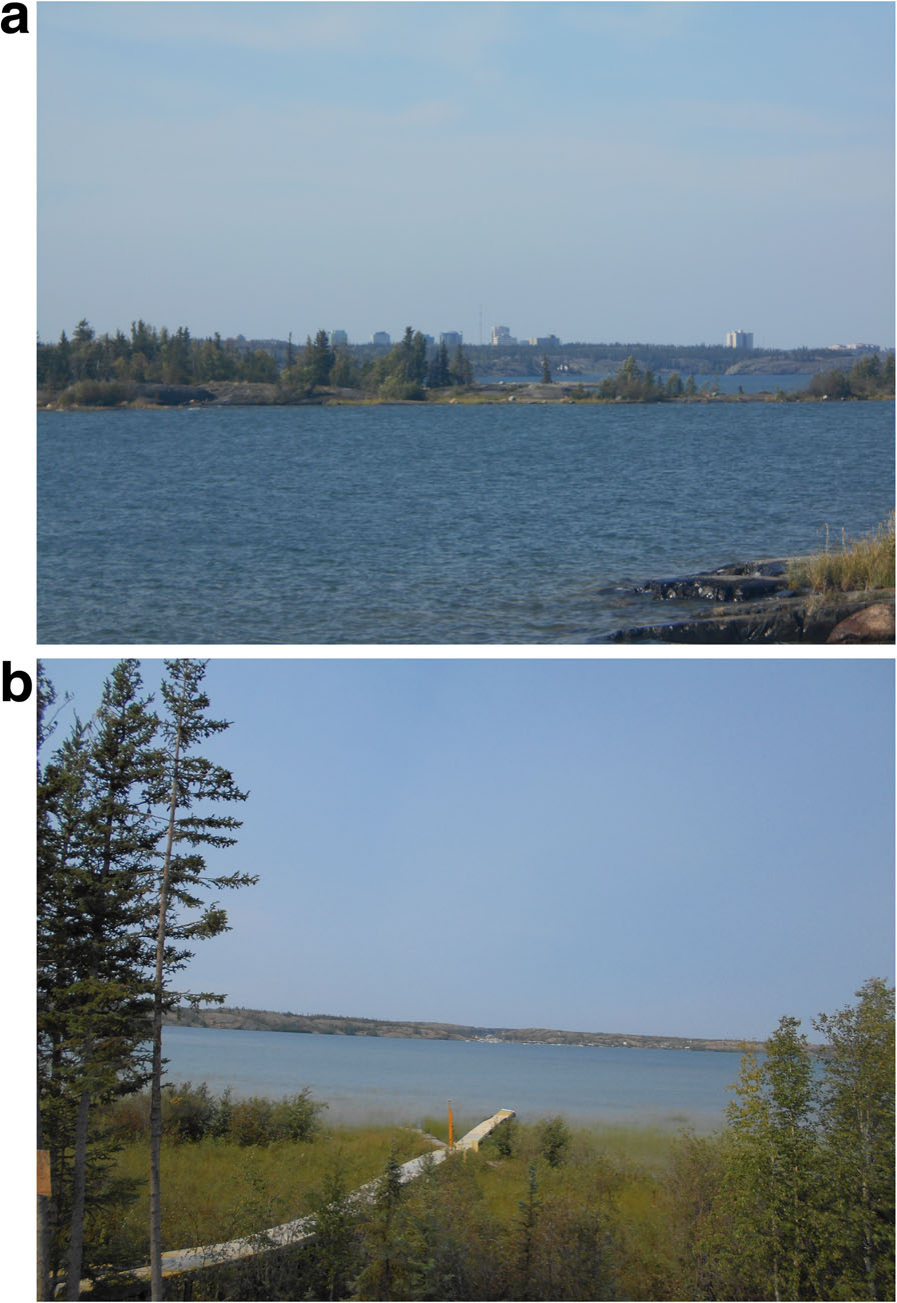
**a** and **b** Youth’s PhotoVoice images of ‘water’ denoted as both a health priority and issue. The first photo (**a**) was taken from Dettah and the second photo (**b**) was taken from Ndilo (with the former Giant Mine gold mine shown in the distance)

The youth analyzed their photos collectively during the on-the-land workshop using the PhotoVoice method developed by Wang and Burris [42]. As a group, the youth discussed photos and cited common examples in support of their analysis. They deliberated about complex topics and reasoned whether images were health issues or priorities. In the discussion below, the youth demonstrated that their thought processes included connections beyond beyond those illustrated in Fig. 3:

Laurie-Ann: *Is water a health priority or issue?*

Youth 4: *Health issue.*

Youth 5: *Health priori… no health issue.*

Youth 6: *That’s what I thought too.*

Youth 7: *Because there’s arsenic.*

Youth 4: *There’s arsenic [from] the Giant Mine.*

Laurie-Ann: *So that’s affected the water. How does that affect your health?*

Youth 5 & 6: *We swim in it. / We drink it.* [at the same time].

Youth 6: *Because we drink it.*

Youth 8: *You can drink it and get poisoned from it or something.*

**Fig. 4:**
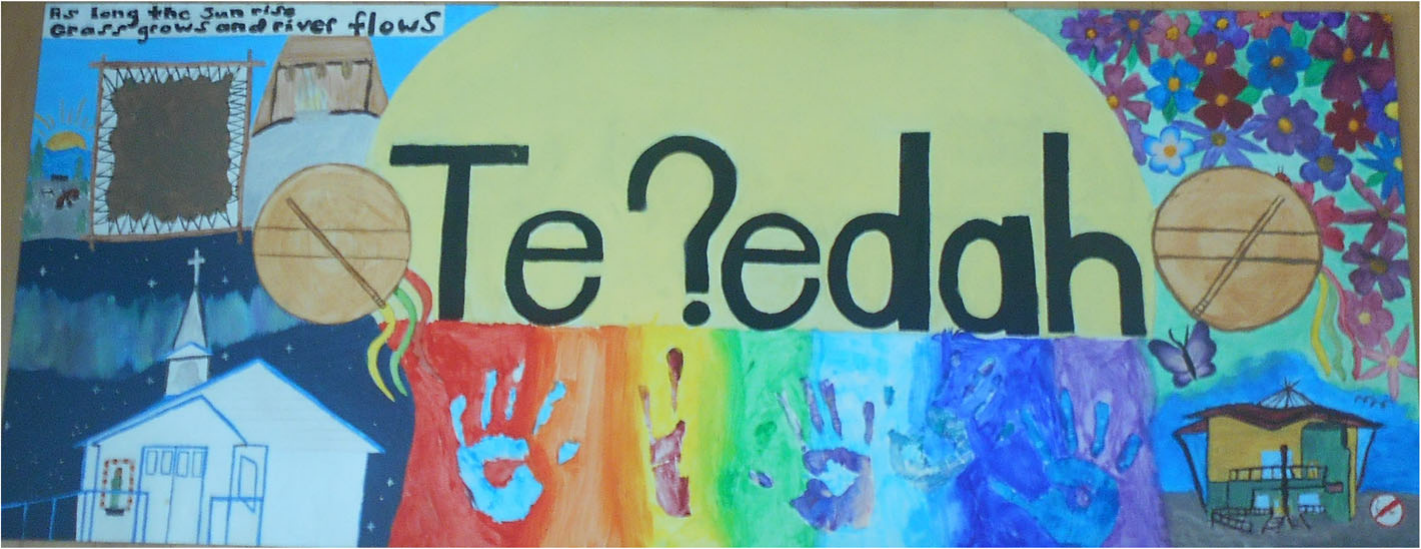
Youth’s mural representing Dettah as a healthy community

**Fig. 5:**
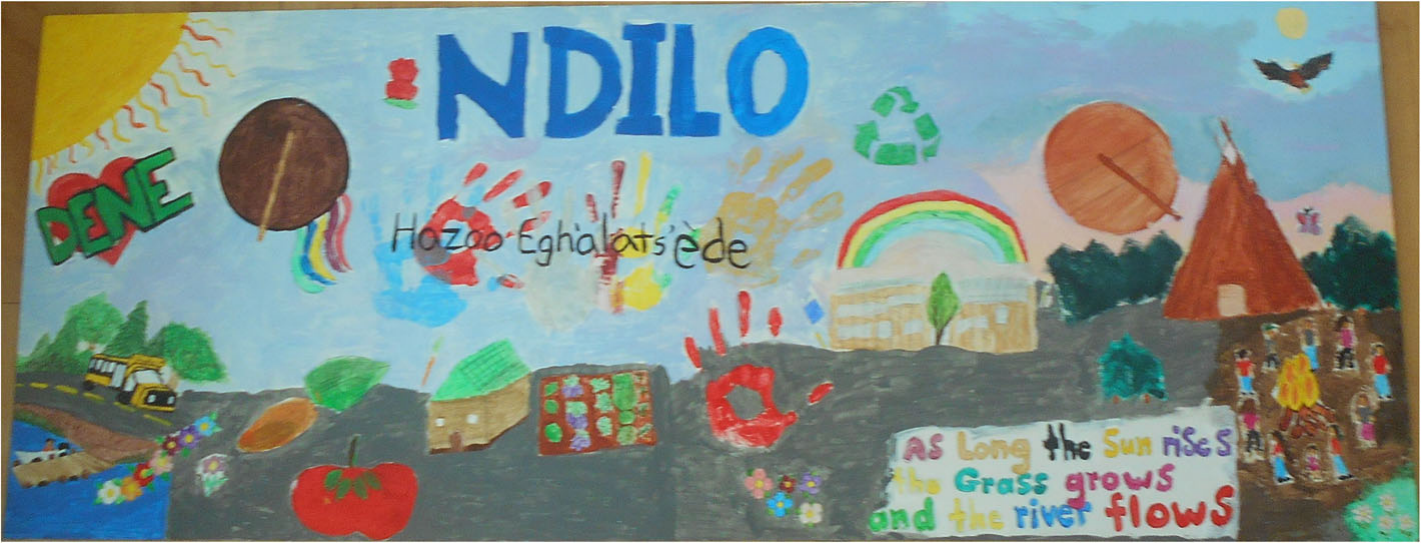
Youth’s mural representing Ndilo as a healthy community

Fifteen youth participated in painting the mural art (Figs. 4 and 5) and 11 youth collectively analyzed the murals and brainstormed 29 mural element depictions. They grouped the elements together and categorized them into themes as they discussed the intention behind each element. They prioritized characteristics most vital for a healthy community by voting which elements most represented a ‘healthy community’. The major elements identified were food and trees/land, followed by water and culture (including language), and then school (get-togethers) and family, friendship, and community. The results were reflective of YKDFN culture. Many of the elements were directly related to the land and represented the youth’s ideas of the reciprocity with the land and the importance of giving back to the land, because it provides for us. The other elements reflected the importance of relationships and culture.

Youth agreed that culture encompassed many traditional ways, customs, knowledge, and skills that were based on a harmonious relation with the land as depicted in mural images. For example, both murals contained images of traditional Dene drumming that represented different aspects of cultural identity including belonging, healing, and spirituality. Also, in both murals the youth wrote the words of the treaty made with Canada (*Treaty 8: “As long as the sun shines, grass grows and the rivers flow…”)* [47] as they recognized their positionality in Canada and upholding the treaty is part of a healthy larger community. They also felt language was a part of culture and purposefully included the local language dialect of Wiiliideh when writing the traditional spelling of the communities. Youth took initiative to demonstrate the heart of the Dene culture by collectively thinking of a phrase that represented their symbols. The youth painted the phrase ‘Hazoo Eghàlats’ède’ in Wiiliideh in a central place on their mural to represent that for a true healthy community “we all work together”. Youth identified all land environment and people relations are connected and must work together harmoniously to achieve positive health.

## Discussion

### Implications of the findings

In Canada, Indigenous Peoples’ views on health encompass not only physical, spiritual, emotional, and mental well-being, but also a positive balance of relational connections between family, community, and land [48]. Each community member has responsibilities to uphold this balance [48], and any issues felt by one cohort, such as youth, are legitimate and realized by the rest of the community [28]. Although much Indigenous literature stresses the relationship between land and Indigenous Peoples from the point of view of Elders and adults [28, 49], less has been documented on youth’s perspectives. Yet this viewpoint is particularly important, as it reflects both current and future health determinants.

Partnering with the YKDFN Wellness Division to combine research with an on-the-land cultural camp and build on YKDFN community strengths primed youth’s role to accurately capture social and structural determinants of YKDFN health. Combining results from several methods, including the five participant methods and field notes of observations from the researcher and research assistants, allowed for the triangulation of data and validated the three main subthemes. In the first subtheme, the youth expressed a relationship between the health of community members and the land. Secondly, the youth envisioned future health solutions as taking place on-the-land, with a distinct youth role in maintaining this relationship through regularly practicing cultural and traditional skills, knowledge, and values. Thirdly, the youth voiced ‘being healthy’ within a community as being related to a symbiotic balance of relationship of land and people, which the youth actively uphold. Together all data supported a clear overall theme where youth identified connection between health and land as a primary determinant to YKDFN health that should be incorporated in future health solutions.

### Youth-identified relationship between health and land

During the research project, YKDFN youth’s perspectives on health meaning, and health priorities and issues, recognized aspects of health specific to the YKDFN traditional way of life. Through visualizations and discussions, the youth continuously tied in a relationship with the land, and ultimately equated a good relationship with the land as a determinant of health. Their viewpoints uniquely underlined the importance of maintaining, preserving, and improving their relationship to the earth because “we survive off the land.” The youth spoke specifically to the importance of actively participating in relationships with Elders and traditional knowledge holders, who pass down knowledge to youth through on-the-land experiences. Relationship to the land was encapsulated by being able to participate in cultural activities, social interactions, and harmonious giving and taking of land elements (such as food and water), taking place on and with the land. The youth felt they had a distinct part in each of these relationships to the land and in the continuation of these connections. These were identified as health priorities. Through their photos, the youth identified health issues that pertained to environmental damage including pollution, modern transportation, and arsenic contamination. Their health solutions were based on identified health priorities and YKDFN strengths, including the community’s traditional ways of developing youth’s connection to the land, such as in traditional teaching and learning.

### Youth’s role in future health solutions and research on-the-land

Overall, youth proposed that they had future roles in health research and community initiatives, with a united vision that the next research project would take place on-the-land. They felt there was a distinct role for youth in promoting health through cultural camps. They saw that a stronger connection to the land, through more cultural activities, meant better health for their community and the land. The youth emphasized the importance of having regular cultural activities encouraging healthy physical activity, traditional eating, mental wellness (stress relief), and continuity of the next generation to grow respectful, long-lasting bonds with people and the environment. They gave specific examples of different ways that they would like to be involved in future research.

### Youth’s understanding of active roles in a symbiotic balance of land and people

The collective approach of this research allowed for youth to delve deeper into underlying factors of ‘being healthy’ within their community through critical thinking of their own and each other’s answers. During the PhotoVoice analysis sessions, the youth compared similarities and differences in their photos and critically assessed how the health priority or issue impacted ‘health’ within the community and of the land. Youth shared stories and gave examples to further explain the complex balance between land and health. Youth also directed the information collected by the researchers in the mural, so that the health and land relationship concept was more clearly understood.

The youth felt the images on the mural alone did not sufficiently capture their vision of a healthy community. They consequently initiated working with community knowledge holders to incorporate the phrase ‘Hazoo Eghàlats’ède’ on their mural to represent that for a true healthy community “we all work together”. The intention behind this statement goes beyond people and emphasized reciprocity. ‘Hazoo Eghàlats’ède’ meant that all the elements in a community (people, land, water, culture, and animals) collectively and actively generate a space that is ‘healthy’. The youth felt that future health initiatives must build on the youth’s relationship with the land, which they must actively uphold, to consequently improve health.

### Overall theme of connection to the land as a determinant of health

YKDFN youth’s perspectives were integral to identifying connection to the land as a major structural determinant of Indigenous health. Learning cultural skills alongside this research built upon the community’s strengths, encouraged thinking about traditional health knowledge, and supported youth’s traditional leadership role in health initiatives. This corresponds to other health research where increased Indigenous control of research creates projects, based on Indigenous assets, to overcome negative health outcomes in a decolonizing fashion [50]. Revitalizing youth’s connection to the land has positive health impacts that reach beyond the direct players of the youth and land, and extend to their family, communities, and surrounding environment. Balanced ecosystems [51] and ownership of, and access to, traditional land has been recognized as improving health status for Indigenous peoples in other areas of the world [52]. YKDFN youth identified that in addition to traditional land access being an Indigenous health determinant, there must be a connection to the land that is upheld by many relations, including youth. Land connection is the basis for ‘Hazoo Eghàlats’ède’ and health benefits being passed down to the next generation in future health solutions.

### Limitations

Not all participants took part in every data collection method due to unforeseen circumstances of personal emergencies, weather interruptions, and scheduling conflicts. The data, however, seems to reflect a good representation of the group as many of the identified theme issues appeared in all of the data collection results. Nonetheless, the insights gained through this research only reflect the thoughts and perspectives of a limited number of youth and are not necessarily transferable to other Dene youth or generalizable to Indigenous youth elsewhere.

## Conclusion

YKDFN youth provided invaluable insights on their perspectives of the important social and structural determinants of health. The youth highlighted their role in influencing future health research and agency to address issues. Working with the community from an asset-based approach permitted research to take place within an on-the-land camp. The localized on-the-land setting and the variety of research methods opened the door for youth’s perspectives to be clearly understood in-depth. Youth participants identified health issues and priorities that extended beyond community life and incorporated cultural components linked to the land. The youth suggested relevant ways to address health issues based on YKDFN strengths already existing in the community, which included youth in key roles. The YKDFN youth’s perspectives for addressing health issues, prioritizing health items, and planning health initiatives emulates the literature indicating that First Nations Peoples must utilize their own culture to reclaim good health outcomes [53]. The YKDFN youth emphasized the importance of building YKDFN culture, community relations, and traditional knowledge transfer through a connection to the land to increase positive health outcomes. Youth considered social and structural determinants of health that were relevant and meaningful to the YKDFN, as their shared examples and definitions were rooted in YKDFN traditions, culture, and worldview.

Projects like this emphasize the importance of involving youth in health initiatives that support them in identifying and addressing health gaps and finding culturally appropriate solutions built on community strengths. The youth highlighted the importance of creating a healthy community through a collective effort based on traditional ways of being. Whether youth gave their perspective on health issues and priorities, agency in health, or health meaning, there was a strong and recurrent underlying theme that revitalizing youth’s connection with the land improves Indigenous youth and community health. Capturing and incorporating youth’s understandings of health sets the stage for future health research that is more culturally appropriate and relevant, and consequently aids in more effective intervention delivery by addressing unique social and structural determinants of Indigenous health.

Our research illustrates the relationship between structural context and Indigenous Peoples’ health, and the need to incorporate this determinant into future health solutions. In line with other research on Indigenous Peoples’ connection with the land [11, 54], we recommend projects with Indigenous communities incorporate land components and the localized strengths of the Indigenous group. Through this research and others [30, 55], First Nations youth have been shown to have multiple active capacities and trusted, representative insights to offer in health initiatives. Moving forward, future research and health programs attempting to address health disparities in Indigenous communities should be considerate of an individual community’s strengths and facilitate local interpretations of health, including the relationship between youth, their community’s culture, and the land.

The YKDFN youth who shared their perspectives strongly exemplify both unique Indigenous worldviews on health solutions based on community strengths and the role of Indigenous youth in defining health meaning and priorities. This supports an overall evolving recognition that Indigenous Peoples’ worldviews and approaches offer distinctive, innovative solutions to colonial health effects when integrated into the foundation of health programs [56].

## Abbreviations

CPBR: Community-Based Participatory Research
YKDFN: Yellowknives Dene First Nations
